# Appropriate Use Criteria–Driven Quality Improvement for PCI in Stable Ischemic Heart Disease: A Multimodal Intervention

**DOI:** 10.1016/j.jaccas.2025.105718

**Published:** 2025-10-07

**Authors:** Samer Kabbani, Kristan D. Langdon, Susanne A. Latham, Mercedes D. Hegler, Tanisha N. Wilcots

**Affiliations:** a Emory Hillandale Hospital, Lithonia, Georgia, USA; b Wellstar West Georgia Medical Center, Cardiology Department, LaGrange, Georgia, USA; c Piedmont Eastside Medical Center, Snellville, Georgia, USA

**Keywords:** appropriate use criteria, percutaneous coronary intervention, quality improvement, stable ischemic heart disease

## Abstract

**Background:**

Appropriate use criteria (AUC) for coronary revascularization in patients with stable ischemic heart disease (SIHD) promote guideline-directed medical therapy, objective ischemia testing, and symptom-driven percutaneous coronary intervention (PCI). Despite national improvements, elevated rates of “rarely appropriate” (RA) PCI persist.

**Project Rationale:**

Benchmarking at a community hospital revealed suboptimal performance on the AUC metric for RA PCI in patients with SIHD, with an average rate of 6.96% and a peak of 12.00%. This reflected gaps in documentation, ischemia assessment, and adequate medical therapy, highlighting the need for a performance improvement initiative.

**Project Summary:**

A multimodal intervention was implemented, integrating 4 key components: targeted education on AUC requirements, embedding AUC data fields into precatheterization templates, monthly case reviews, and promoting a “physiology-first” culture, with fractional flow reserve when noninvasive evidence was absent. The primary endpoint was the proportion of elective SIHD PCI cases classified as RA. After implementation, the average RA PCI rate decreased from 6.96% to 0% by the fourth quarter of 2023 and was sustained for 6 consecutive reporting quarters, through the first quarter of 2025.

**Take-Home Message:**

Integrating AUC elements directly into clinical workflows supported by targeted education, a physiology-first approach, and continuous feedback can eliminate RA PCI in SIHD and sustain performance improvement over time.

## Graphical Abstract

**Figure:**
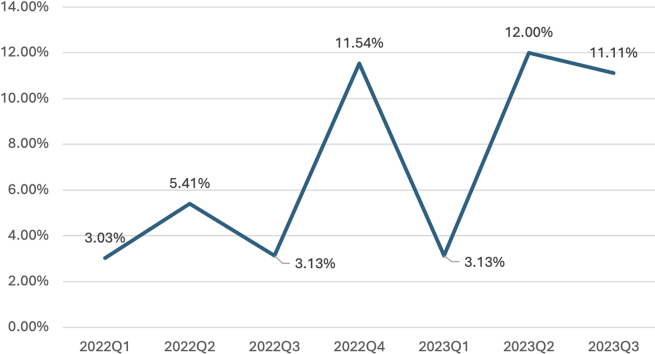

Over the past decade, the American College of Cardiology, in collaboration with multisociety partners, has developed appropriate use criteria (AUC) for coronary revascularization to guide patient selection for percutaneous coronary intervention (PCI) in stable ischemic heart disease (SIHD).^1^ Key principles of the AUC include the following:

1. Symptom relief as the primary goal of PCI.
2. Consistent use of guideline-directed medical therapy.
3. Objective evidence of ischemia and/or lesion-level physiology (eg, fractional flow reserve [FFR]) prior to revascularization.
4. Escalation of antianginal therapy before PCI in most nonurgent cases.

The 2017 AUC revision refined appropriateness categories (“appropriate,” “may be appropriate,” “rarely appropriate”), expanded physiologic assessment, and clarified documentation elements critical for accurate case mapping.^1^^,^^2^ National registry data link AUC implementation, audit-and-feedback, and payer incentives with substantial reductions in nonacute PCI volumes and “inappropriate”/“rarely appropriate” classifications.^3^, ^4^, ^5^ However, persistent interhospital variation and documentation gaps remain barriers to optimal AUC adoption.^6^

**Take-Home Messages**

• Eliminating “rarely appropriate” PCI for SIHD is feasible with embedded documentation and feedback systems.
• Ischemia assessment with guideline-directed medical therapy prior to catheterization laboratory referral aligns with best practices.
• Sustained improvement requires integration of AUC principles into everyday workflow and continuous performance monitoring.

## Project Rationale

**Figure 1:**
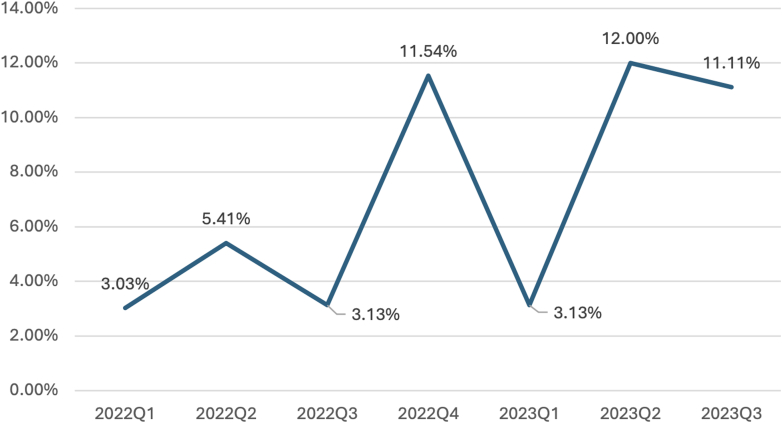
Rates of Stable Ischemic Heart Disease PCI Classified as “Rarely Appropriate” Shown are data from Q1 2022 to Q3 2023, with a peak reported rate reaching 12%. AUC = appropriate use criteria; PCI = percutaneous coronary intervention.

National Cardiovascular Data Registry (NCDR) CathPCI Registry benchmarking at a community hospital in 2023 identified performance below the 10th percentile for the AUC “rarely appropriate” (RA) category on the SIHD PCI metric. The rolling 4-quarter rate was 6.96%, peaking at 12.00% in the second quarter (Q2) of 2023 (Figure 1). Contributing system-level barriers included fragmented documentation, inconsistent stress test reporting, and limited preprocedural review. These factors are known to hinder AUC compliance and accurate NCDR CathPCI mapping.

## Project Description

In fiscal year 2024, a performance improvement initiative was designed to close these gaps and align practice with the 2017 SIHD AUC. The project objectives were to

1. Reduce the proportion of elective PCI cases classified as RA to 0%, with sustained improvement.
2. Standardize precatheterization documentation for all essential AUC data elements, symptom status, antianginal therapy, ischemia assessment, risk classification, and coronary anatomy.
3. Increase use of FFR or instantaneous wave-free ratio (iFR) to adjudicate lesion significance when noninvasive findings were equivocal.

## Intervention Components

**Figure 2:**
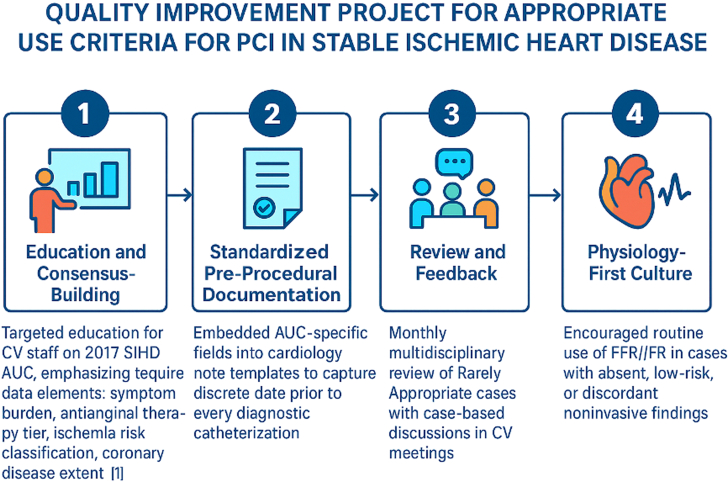
Modalities Used to Improve Quality of Care The 4 modalities used to eradicate “rarely appropriate” PCI for stable ischemic heart disease included 1) targeted education monthly, 2) standardized preprocedural documentation, 3) monthly case review and feedback to the operators and referral physicians, and 4) encouraging the routine use of physiologic assessment of lesions prior to intervention. AUC = appropriate use criteria; CV = cardiovascular; FFR = fractional flow reserve; iFR = instantaneous wave-free ratio; PCI = percutaneous coronary intervention; SIHD = stable ischemic heart disease.

The initiative consisted of 4 components:

• Education and consensus: Targeted training on 2017 SIHD AUC, including data element requirements for the 2017 AUC guidelines.
• Standardized documentation: Embedded AUC-specific fields in preprocedural documentation to ensure discrete capture of required information prior to every diagnostic catheterization.
• Review and feedback: Monthly multidisciplinary case review of RA PCI, coupled with real-time NCDR data monitoring and operator feedback.
• Physiology-first culture: Routine encouragement of FFR/iFR in borderline or discordant cases to guide PCI (Figure 2).

## Project Deliverables

The project delivered updated cardiology documentation templates with embedded AUC-specific fields to ensure complete and standardized data capture. A structured educational program was implemented to train staff on AUC standards and their practical application. A formalized monthly case review process with an integrated feedback loop was established to promote continuous quality improvement. In addition, a real-time performance dashboard was developed to track and report NCDR Cath-PCI metrics.

## Project Outcomes

**Figure 3:**
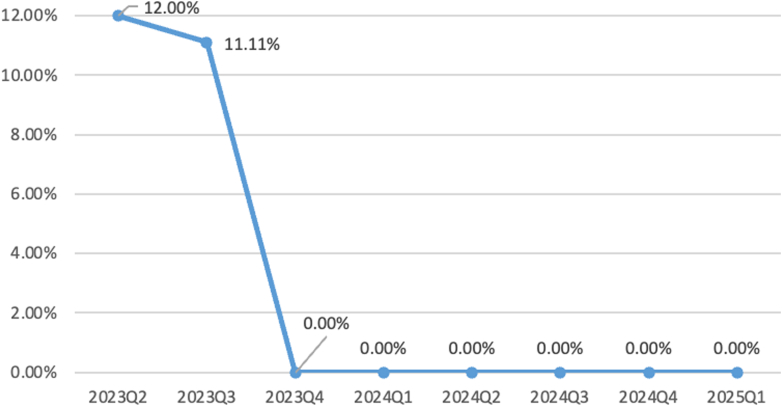
Rates of “Rarely Appropriate” PCI for Stable Ischemic Heart Disease There was a drop in “rarely appropriate” PCI rates in SIHD from 12% to 0%, with sustained improvements over a 1-year period from Q1 2024 to Q1 2025. PCI = percutaneous coronary intervention; SIHD = stable ischemic heart disease.

The primary outcome was a reduction in the rolling 4-quarter RA PCI rate from 6.96% in Q2 2023 to 0% by Q4 2023. This improvement was sustained, with a 0% rate maintained for 6 consecutive NCDR reporting quarters, through Q1 2025 (Figure 3). Documentation completeness reached nearly 100% across all AUC data elements. The use of FFR/iFR did not change significantly from year to year, reflecting better ischemia screening prior to catheterization laboratory referral. Analysis of procedural data revealed a decrease in the total number of yearly PCI procedures, from 236 in 2023 to 200 in 2024. This reflected a change in practice, with more use of guideline-directed medical therapy rather than ad hoc PCI for patients with stable CAD.

## Discussion

Previous quality improvement initiatives emphasized dissemination of updated AUC, registry-based benchmarking, and policy-driven incentives to improve PCI appropriateness in SIHD.^7^, ^8^, ^9^ Although these approaches provided important frameworks, their translation into consistent practice remained variable. Our intervention extended prior work by embedding AUC principles directly into clinical workflows through targeted provider education, standardized documentation, structured case review with feedback, and, most importantly, a physiology-first approach. Unlike earlier efforts, which often relied on passive dissemination or retrospective monitoring, our strategy enabled real-time data capture, prospective decision support, and iterative feedback. This integration not only improved adherence to the 2017 SIHD AUC but also fostered a culture of accountability and ischemia-guided decision-making, resulting in measurable gains in appropriateness and sustained improvements in practice.

Historically, patients were frequently referred to the catheterization laboratory based on elevated calcium score on computed tomography, without documented evidence of ischemia on stress testing. Moreover, patients with history of coronary artery disease or coronary artery bypass grafting who presented with anginal symptoms were referred by their physicians directly to the catheterization laboratory for procedures. In such cases, the interventional cardiologist often performed ad hoc PCI without proof of ischemia or adequate antianginal therapy. This practice is classified as “rarely appropriate” by the NCDR. After the implementation of our performance improvement initiative, the referral pattern changed. Patients were required to have stress testing, and antianginal therapy was augmented before referral to the catheterization laboratory. This initiative was associated with a reduction in the total number of PCI procedures, from 236 in 2023 to 200 in 2024, and it resulted in a sustained drop of “rarely appropriate” PCI to 0%.

## Conclusions

A structured, multimodal approach can align elective PCI practice with AUC standards, yielding sustained quality improvement.

## Funding Support and Author Disclosures

Wellstar Health Care System supported the study for abstract presentation, including travel, accommodation, and meals. The authors have reported that they have no relationships relevant to the contents of this paper to disclose.
